# What Comes from Cytology Diagnosis: A Comprehensive Epidemiological Retrospective Analysis of 3068 Feline Cases

**DOI:** 10.3390/vetsci12070671

**Published:** 2025-07-17

**Authors:** Paula Brilhante-Simões, Ricardo Lopes, Leonor Delgado, Ana Machado, Augusto Silva, Ângela Martins, Ricardo Marcos, Felisbina Queiroga, Justina Prada

**Affiliations:** 1 INNO Veterinary Laboratories, R. Cândido de Sousa 15, 4710-300 Braga, Portugal; paulabrilhante@inno.pt (P.B.-S.); leonordelgado@inno.pt (L.D.); anamachado@inno.pt (A.M.); augustosilva@inno.pt (A.S.); 2 Department of Veterinary and Animal Sciences, University Institute of Health Sciences-CESPU (IUCS-CESPU), 4585-116 Gandra, Portugal; lopes.rmv@gmail.com; 3 Department of Veterinary Sciences, University of Trás–os–Montes e Alto Douro (UTAD), 5000-801 Vila Real, Portugal; fqueirog@gmail.com; 4 UNIPRO-Oral Pathology and Rehabilitation Research Unit, University Institute of Health Sciences-CESPU (IUCS-CESPU), 4585-116 Gandra, Portugal; 5 Animal and Veterinary Research Centre (CECAV), Associate Laboratory for Animal and Veterinary Sciences (AL4AnimalS), University of Trás-os-Montes e Alto Douro (UTAD), 5000-801 Vila Real, Portugal; angela@utad.pt; 6 Cytology and Hematology Diagnostic Services, Laboratory of Histology and Embryology, Department of Microscopy, ICBAS-School of Medicine and Biomedical Sciences, University of Porto (U.Porto), Rua de Jorge Viterbo Ferreira, 228, 4050-313 Porto, Portugal; rmarcos@icbas.up.pt; 7 Animal Morphology and Toxicology Team, CIIMAR, Interdisciplinary Centre of Marine and Environmental Research, University of Porto (U.Porto), 4450-208 Matosinhos, Portugal; 8 Center for the Study of Animal Sciences, CECA-ICETA, University of Porto (U.Porto), 4200-465 Porto, Portugal

**Keywords:** anatomic location, cancer, clinical pathology, cytology, diagnosis, cat, epidemiology, neoplasms

## Abstract

This study evaluated diagnostic trends and the overall utility of cytology in feline patients through the analysis of a large, multicentric dataset from Portugal. A retrospective review of 3068 cytological cases from 130 veterinary practices was conducted, with samples categorised by anatomical location and lesion type. Diagnostic outcomes were statistically assessed, revealing an overall success rate of 66.20%. The highest diagnostic yields occurred in fluid samples (83.48%), glandular tissues (76.67%), and mucous membranes (75.81%), followed by organ-based samples (67.79%), miscellaneous tissues (66.98%), cutaneous/subcutaneous nodules (62.16%), and lymph nodes (57.93%). Neoplastic lesions showed age-associated prevalence, being more common in older cats, with epithelial and melanocytic lesions more frequent in females and round cell/mesenchymal lesions predominating in males. Non-diagnostic samples (33.80%) primarily resulted from insufficient cellularity or suboptimal quality, though no significant correlation existed between diagnostic success and clinical setting. This study underscores that cytology remains a fundamental diagnostic tool in feline medicine, particularly when combined with proper sampling techniques and complementary diagnostic methods, and reinforces its value in clinical decision-making, thereby supporting its broader utilisation in routine veterinary practice.

## Simple Summary

Cytology is a minimally invasive and cost-effective diagnostic method used in veterinary medicine to examine cells from tissues, fluids, and other body structures. This study analysed over 3000 cases from cats across Portugal to evaluate the effectiveness of cytology in diagnosing various conditions. The highest diagnostic success rates were observed in fluids and gland tissues, while samples from skin lesions and lymph nodes were more challenging. Tumours were more frequent in older cats, while younger cats often had inflammation. Some tumours, such as epithelial cancers, appeared more in females, whereas other types, like mesenchymal cancers, were more common in males. Poor-quality or incomplete samples often resulted in inconclusive findings, highlighting the need for proper collection and preparation. This research demonstrates the vital role of cytology in detecting diseases early, guiding treatment decisions, and improving the overall care of feline patients. With meticulous sample handling and integration with other diagnostic methods, cytology remains an invaluable tool in veterinary medicine.

## 1. Introduction

Cytology is an essential diagnostic tool in veterinary medicine, widely recognised for its ease of execution, minimally invasive nature, and cost-effectiveness [1,2,3,4]. It provides a rapid and accessible means of assessing the cellular composition of lesions [4,5], thereby facilitating the diagnosis and classification of a broad spectrum of pathological conditions. Owing to its versatility, cytology is routinely employed in the evaluation of cutaneous and subcutaneous masses, cystic structures, ulcerative lesions, and fistulous tracts. Furthermore, it plays a pivotal role in the examination of organ samples and body fluids, including effusions, cerebrospinal fluid (CSF), urine, and synovial fluid [2,3,4,6,7,8,9,10,11].

A variety of techniques are available for collecting cytological samples, including fine-needle aspiration (FNA), fine-needle non-aspiration, impression smears, swabs, and scrapings [12,13,14]. The selection of technique largely depends on the lesion’s characteristics, anatomical location, and the practitioner’s preference [13]. Despite its numerous advantages, cytology has inherent limitations [15,16], primarily due to the absence of tissue architecture, which is critical for differentiating between benign and malignant conditions [17]. Despite these limitations, cytology often provides sufficient information for a definitive diagnosis. In instances where a specific conclusion is elusive, the insights gained by cytology can direct further diagnostic efforts to achieve a conclusive diagnosis [7,18,19,20].

The diagnostic accuracy of cytology is influenced by multiple factors, including sample quality, cellular preservation, and preparation techniques. Inadequate sample selection, suboptimal collection methods, and improper slide preparation can lead to non-diagnostic or inconclusive results [2,18,21]. Therefore, meticulous attention to sample acquisition and preparation is essential to maximise the clinical utility of cytological evaluation. Numerous studies in companion animals have highlighted the diagnostic value of cytology [1,5,6,7,11,15,19,20,22,23,24,25]. In feline medicine, cytology is extensively used to diagnose neoplastic and inflammatory conditions affecting cutaneous and subcutaneous lesions [7,11,26], internal organs such as the spleen [27] and urinary tract/kidney [28], bone [25], skeletal muscle [29], oral lesions [1,24], the lymphatic system [5,15], and the mammary gland [30].

This study conducts a comprehensive retrospective analysis of cytological diagnoses in cats over a seven-year period, encompassing 3068 cases from veterinary practices throughout Portugal. To the authors’ best knowledge, this represents the first large-scale investigation into the epidemiological relevance of cytology for feline diagnostics in the country. By examining diagnostic trends and outcomes, this study highlights the fundamental role of cytology in veterinary medicine, reinforcing its significance as a critical tool for early disease detection and informed clinical decision-making.

## 2. Materials and Methods

### 2.1. Data Collection, Sampling, and Diagnostic Procedures

Cytology samples were submitted to INNO Veterinary Laboratories (Braga, Portugal) over a seven-year period. The samples were submitted by a total of 130 veterinary practices located across all districts of mainland Portugal and from the Insular Autonomous Regions.

The vast majority of cytological samples received were submitted unstained. These were processed in-house and stained using a Romanowsky-type stain (Hemacolor^®^, Merck KGaA, Darmstadt, Germany) with an automated RAL Stainer (RAL Diagnostics, Martillac, France), ensuring consistent and reproducible staining quality. The staining protocol followed the manufacturer’s specifications. Pre-stained slides were submitted only occasionally (<5%), but due to the retrospective nature of the study, the exact proportion and details of externally applied staining methods could not be determined.

After staining, all slides were mounted using Entellan™ (Sigma-Aldrich, St. Louis, MO, USA) to preserve the preparations. Microscopic evaluation was performed using a Nikon Eclipse 600 microscope (Nikon Corporation, Tokyo, Japan) equipped with CFI Plan Achromat objectives: 10× (Ref. MRP70100), 20× (Ref. MRL00202), 40× (Ref. MRP70400), 50× (Ref. MRL01502), and 100× oil immersion (Ref. MRL01903). No digital scanning methods were employed in this study; all evaluations were conducted using direct light microscopy. Cytological interpretation was performed by two experienced clinical pathologists, each with over 30 years of continuous practice in diagnostic cytopathology. Although not board-certified diplomates, both professionals have extensive training and a well-established record in the field, ensuring a high level of diagnostic consistency and reliability throughout the study. The same two evaluators remained consistent throughout the seven-year study period (2011–2016). All samples were initially evaluated independently; in cases of uncertainty or inconclusiveness, the pathologists discussed the findings collaboratively to reach a consensus diagnosis. In cases of diagnostic uncertainty, the remaining co-authors, also experienced in cytological interpretation, were involved in the review process to reach a broad consensus. Additionally, in selected cases, samples were submitted to external specialised veterinary laboratories for second-opinion evaluation, thereby supporting the accuracy and consistency of the final diagnosis. Fluid samples were submitted either in plain tubes or in tubes with EDTA. These were transported under refrigeration, with sample stability ensured through controlled temperature conditions maintained by the laboratory’s dedicated logistics team. In cases of low cellularity, centrifugation was performed upon arrival to concentrate cellular components prior to smear preparation. Due to the retrospective design of the study, data on the specific sample collection techniques were not available (e.g., fine-needle aspiration, non-aspiration, and touch imprint). However, all samples were collected in accordance with the sampling guidelines and best practice recommendations provided by the laboratory to all participating clinics.

Accompanying each sample was a laboratory request form that provided essential clinical information, including breed, sex, age, clinical suspicions or signs; the anatomical location of the sample; and the analyses requested. The age of the animals was categorised into seven groups following a previous methodology [31].

Samples were categorised by anatomical location as follows: subcutaneous and cutaneous locations; lymph nodes; glandular tissues (including mammary glands, endocrine glands, and pancreas); fluids (comprising joint fluid, urine, and transtracheal or bronchoalveolar washes); miscellaneous (including intra-abdominal and intrathoracic masses, mediastinum, mesentery, bone, ear, testicle, and penis); mucous membranes (covering vaginal, oral, ocular, nasal, and anal locations); and organs (including the lungs, kidneys, liver, and spleen). Samples requiring additional diagnostic modalities, such as bone marrow myelograms, thoracic, abdominal, and pericardial effusions, or cerebrospinal fluid, were excluded from the study.

Furthermore, samples were classified into **diagnostic categories**—neoplastic (comprising epithelial, spindle cells, round cells, and melanocytic lesions) [28,32]; inflammatory (subdivided into infectious, suppurative, pyogranulomatous/granulomatous, eosinophilic, lymphocytic, plasmocytic, and those of unknown origin) [12,28,32,33]; non-neoplastic/non-inflammatory (including cystic, degenerative and haemorrhagic lesions, hyperplasia, corticosteroid-induced hepatopathy, and extramedullary haematopoiesis, among others) [34,35]; and the “other” category, which included normal samples and vaginal cytology for oestrous cycle staging. The designation “infectious” was applied when there was cytological evidence of infectious organisms, regardless of the predominant inflammatory cell type. **Non-diagnostic categories** were designated as indicative (providing suggestive information towards a potential diagnosis, e.g., when it is not possible to determine the subtype of neoplasia) and inconclusive (characterised by low cellularity, haemodilution, poor sample quality, or destroyed cells) [36,37].

The classifications were further simplified into two overarching categories: **neoplastic** and **non-neoplastic**, with the latter encompassing inflammatory and non-neoplastic/non-inflammatory processes and other sample types.

This study is based exclusively on cytological diagnoses. Although histopathological confirmation may have been performed in some individual cases as part of routine clinical follow-up, such data were not systematically collected or analysed, and histology was not used to validate or override the cytological findings presented. The primary objective of this study was not to assess the diagnostic accuracy of cytology against histopathology, but rather to provide a large-scale epidemiological overview of cytological utilisation and diagnostic outcomes in feline patients.

### 2.2. Statistical Analysis

Statistical evaluations were conducted using JMP^®^, version 14.3 (SAS Institute, Cary, NC, USA, 1989–2023), DATAtab^®^ (DATAtab e.U., Graz, Austria, 2024), and MedCalc^®^ Statistical Software version 20.006 (MedCalc Software Ltd., Ostend, Belgium). Proportional differences were examined using the Chi-Square test (χ^2^), utilising Fisher’s exact test when expected frequencies fell below five. In analysing breed predisposition, univariable logistic regression was utilised to determine the odds of neoplastic versus non-neoplastic conditions, considering potential biases in breed representation. A significance threshold of *p* ≤ 0.05 was considered.

## 3. Results

A total of 3068 animal samples were received for analysis. Of these, 1597 samples (52.05%; 95% CI: 50.3–53.8%) were from females and 1471 (48.0%; 95% CI: 46.2–49.7%) were from males.

Information on animal breed was absent in 590 (19.24%) of the request forms. From the available data (*n* = 2478), 13 different breeds were identified. The breakdown included 2018 Domestic Shorthair (DSH) (65.80%), 247 Persian (8.05%), 141 Siamese (4.60%), 30 Norwegian Forest (0.98%), 10 Sphynx (0.33%), 9 Main Coon (0.29%), 6 Turkish Angora (0.20%), 6 Burmese (0.20%), 4 British Shorthair (0.13%), 2 Scottish Fold (0.07%), 2 Somali (0.07%), 1 Bobtail (0.03%), and 1 Chartreux (0.03%).

Age data were available for 2625 animals (85.56%), while 443 request forms (14.44%) did not specify the animals’ age. The age distribution ranged from ≤1 year (6 months) to 23 years, with a median age of 8 years (interquartile range: 4–12 years). A total of 13.2% (95% CI: 12.0–14.6; *n* = 347) were classified as kittens, 6.2% (95% CI: 5.4–7.2; *n* = 163) as young, 10.0% (95% CI: 8.9–11.2; *n* = 263) as young adults, 16.0% (95% CI: 14.6–17.4; *n* = 419) as adults, 19.1% (95% CI: 17.7–20.7; *n* = 502) as mature adults, 30.0% (95% CI: 27.8–31.3; *n* = 775) as seniors, and 5.9% (95% CI: 5.1–6.9; *n* = 156) as geriatrics.

### 3.1. Diagnostic Yield

In this study, of the 3068 cytological samples analysed, 2031 (66.20%) yielded a definitive diagnosis, while 1037 (33.80%) were non-diagnostic. Within the diagnostic outcomes, 714 samples (23.27% of the total) were identified as neoplastic, 691 samples (22.52%) showed inflammatory changes, 439 samples (14.31%) were categorised as non-neoplastic and non-inflammatory, and 187 samples (6.1%) fell into the “other” category. On the non-diagnostic side, 564 samples (18.38%) provided indicative findings that suggested a possible diagnosis, and 473 samples (15.42%) were inconclusive, yielding neither specific nor suggestive results.

**Figure 1:**
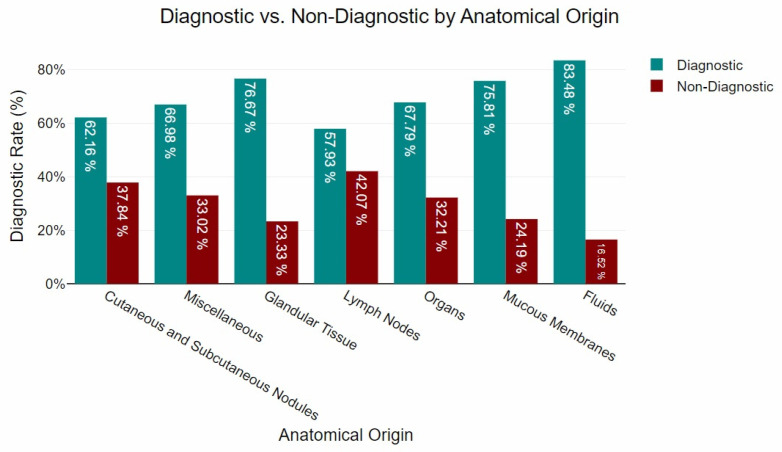
Diagnostic yield by anatomical origin.

Based on the anatomical origin, the majority of the samples (*n* = 1094) were obtained from cutaneous and subcutaneous nodules. Of these, 62.16% contained sufficient cytological features to support a definitive interpretation. A total of 444 samples were collected from internal organs, with 67.79% meeting the criteria for cytological assessment. Lymph node aspirates comprised 274 samples, of which 57.93% were considered cytologically interpretable. Glandular tissues were sampled in 138 cases, yielding a high proportion of interpretable results (76.67%). Mucosal tissues contributed 257 samples, with 75.81% deemed adequate for diagnostic evaluation. Among the 142 samples classified as miscellaneous, 66.98% were suitable for interpretation. Finally, fluid samples (*n* = 96) showed the highest proportion of interpretable material, at 83.48%. The distribution of sample adequacy by anatomical site is illustrated in Figure 1.

### 3.2. Neoplastic Versus Non-Neoplastic Diagnoses

The incidence of neoplastic processes was first analysed in relation to epidemiological factors (sex, breed, and age), followed by an assessment based on anatomical location.

#### 3.2.1. Distribution of Neoplastic and Non-Neoplastic Lesions by Sex, Breed, and Age

A total of 2031 samples were evaluated, with 1083 from females and 948 from males. Among the female samples, 392 (54.90%) were diagnosed as neoplastic, whereas 691 (52.47%) were non-neoplastic. In the male group, 322 samples (45.10%) were neoplastic, and 626 (47.53%) were non-neoplastic. Overall, neoplastic lesions accounted for 714 cases (35.16%) of the total sample population, while non-neoplastic cases represented the majority, with 1317 cases (64.84%). The Chi-Square test of independence between sexes did not indicate a significant association between male and female neoplastic lesions (*p* = 0.294), indicating a consistent frequency of neoplastic occurrences among the sexes evaluated.

Regarding the breed, no significant association was found between the breed and the occurrence of neoplastic conditions (Chi-Square test, *p* = 0.303), indicating a uniform distribution of neoplastic occurrences across the various breeds evaluated. 

In contrast to sex and breed, age showed a significant association with the incidence of neoplasia (Chi-Square test, *p* < 0.001). In animals aged ≤ 1 year, 10.3% of lesions were neoplastic. This proportion increased to 21.4% in the >1 to ≤2 years group and 24.3% in the >2 to ≤4 years group. These younger animals served as the baseline for comparison, with the ≤1 year group set as the reference (*odds ratio* (OR) = 1).

The likelihood of neoplastic diagnosis increased progressively with age:

- >1 to ≤2 years: OR = 0.97 (95% CI: 0.60–1.55);
- >2 to ≤4 years: OR = 1.14 (95% CI: 0.76–1.70);
- >4 to ≤7 years: OR = 1.39 (95% CI: 1.00–1.93);
- >7 to ≤10 years: OR = 2.68 (95% CI: 1.98–3.62);
- >10 to ≤15 years: OR = 3.73 (95% CI: 2.85–4.88);
- >15 years: OR = 4.90 (95% CI: 3.14–7.66).

Table 1 displays the occurrence of non-neoplastic versus neoplastic lesions according to age group.

#### 3.2.2. Distribution of Neoplastic and Non-Neoplastic Lesions by Anatomical Location

In the analysis, cutaneous and subcutaneous nodules constituted the primary category, encompassing 680 samples, of which 48.09% (*n* = 327) were characterised as neoplastic and 51.91% (*n* = 353) as non-neoplastic. The second largest category, consisting of 142 miscellaneous samples, exhibited a neoplastic incidence of 46.48% (*n* = 66) versus 53.52% (*n* = 77) of non-neoplastic. Glandular tissue, contributing 138 samples, displayed a neoplastic lesion rate of 45.65% (*n* = 63) against 54.35% (*n* = 75) non-neoplastic. Lymph nodes, with 274 samples assessed, showed a lower neoplastic proportion at 33.94% (*n* = 93) versus non-neoplastic (*n* = 181; 66.6%). Organ samples, which totalled 444, had a neoplastic lesion proportion of 29.28% and a non-neoplastic proportion of 70.72% (*n* = 314). Mucous membranes, accounting for 257 samples, primarily consisted of non-neoplastic lesions, with a significantly lower rate of 13.23% and 86.77% (*n* = 223) non-neoplastic. Fluid samples, the smallest group in this study with 96 specimens, demonstrated the lowest incidence of neoplastic conditions, at just 1.04% and 98.96% (*n* = 95) non-neoplastic.

**Figure 2:**
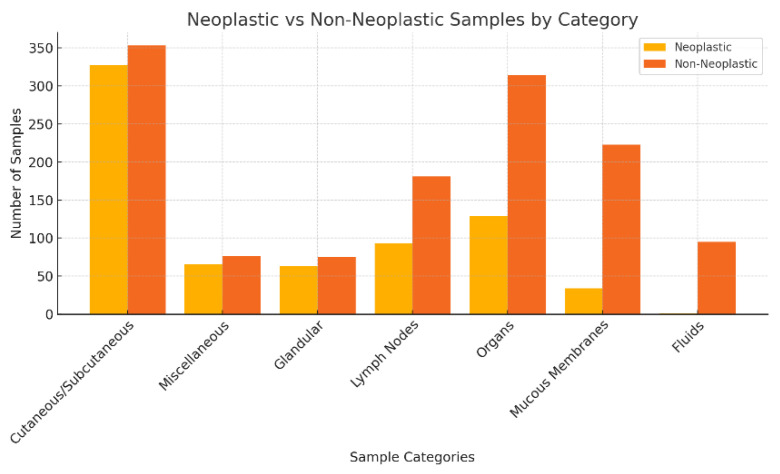
Neoplastic vs. non-neoplastic samples by category.

Figure 2 presents the distribution of neoplastic and non-neoplastic lesions by category in this study.

#### 3.2.3. Neoplastic Diagnosis

In an attempt to determine whether the incidence of different cell types was associated with sex and age within the group of neoplastic diagnoses, Table 2 presents a descriptive summary of the occurrences of various types of neoplastic lesions, categorised according to their nature: round cell, mesenchymal, epithelial, and melanocytic tumours.

In terms of sex, females showed a higher proportion of epithelial tumours (38.01%), whereas mesenchymal lesions were more frequently observed in males (42.86%). Round cell tumours had a similar distribution between sexes (31.12% in females and 34.16% in males), and melanocytic tumours were rare in both groups, accounting for less than 1% of cases. Regarding age, among the 621 cases evaluated, round cell lesions were the most frequent overall (35.99%), followed by mesenchymal lesions (32.49%), epithelial lesions (31.09%), and melanocytic lesions (0.42%). Round cell tumours predominated in animals aged ≤1 year and >1 to ≤2 years, representing over 80% of the neoplasms in these age groups. Conversely, epithelial and mesenchymal lesions became more prevalent in older animals, particularly in the age groups >10 to ≤15 years and >15 years, where epithelial tumours accounted for up to 39.66% of the diagnoses (Table 2).

## 4. Discussion

This study presents a comprehensive retrospective analysis of cytology as a diagnostic tool in feline veterinary medicine over a seven-year period. Analysis of 3068 samples from 130 veterinary practices across Portugal yielded a diagnostic success rate of 66.20%, emphasising the critical relevance of cytology in routine practice. Our findings align with the existing literature on the application of cytology in veterinary medicine, particularly its use in diagnosing neoplastic and non-neoplastic conditions. While cytology is widely recognised for its rapid turnaround and accessibility, several limitations remain, primarily linked to the absence of tissue architecture, sample quality, and diagnostic sensitivity when compared to histopathology [5,28,33,34].

Cytology remains a fundamental tool for the preliminary assessment and classification of lesions, particularly through FNA, which offers valuable insights into cellular composition [23]. However, a significant proportion of samples were classified as non-diagnostic, mirroring results observed in canine studies [38,39]. Contributing factors included insufficient cellularity, haemodilution, and suboptimal slide preparation technique [18,21]. Despite these limitations, cytology has demonstrated high diagnostic concordance with histopathology in specific lesion types, particularly in the identification of round cell neoplasms [5,40,41]. Nonetheless, epithelial and mesenchymal neoplasms exhibited a higher rate of inconclusive cytological diagnoses, reinforcing the recommendation for histopathological confirmation in such cases [42]. It is important to note that, unlike other studies, cytological diagnoses in this research were not confirmed by histopathology, as the primary objective was not to assess the accuracy of cytological examinations [2,15]. Therefore, the identified factors should not be interpreted as determinants of increased diagnostic reliability, but rather as variables that enhance the likelihood of a cytopathologist formulating a clinically meaningful interpretation [2].

The present study revealed significant variations in diagnostic yield across anatomical groupings, reinforcing the influence of sample origin on cytological outcomes. Fluids exhibited the highest diagnostic success rate (83.48%), followed by glandular tissue (76.67%) and mucous membranes (75.81%). These findings align with previous research highlighting the superior cytological yield of fluid-based samples, which generally provide higher cellularity and well-preserved morphology, facilitating interpretation [43,44,45]. Similarly, the high yield observed in mucous membranes and glandular tissue may be attributed to the relative homogeneity of their cellular composition, which enhances diagnostic accuracy [24,25,46,47]. Organs demonstrated a notable diagnostic success rate (67.79%), consistent with previous reports, despite the inherent challenges in obtaining representative samples from these tissues [20,25,48]. Conversely, lower rates were observed in miscellaneous (66.98%), cutaneous and subcutaneous nodules (62.16%), and lymph nodes (57.93%). These findings corroborate previous studies [7,11,49], suggesting that cutaneous and subcutaneous samples, despite being more accessible, are prone to artefacts such as haemodilution, poor cellular preservation, and inflammatory contamination, which can reduce diagnostic accuracy. The diagnostic performance of FNA cytology in lymph node evaluation underscores the challenges associated with differentiating reactive hyperplasia from neoplastic infiltration, as previously documented [5,15,50]. While cytology remains a valuable tool for preliminary assessment, histopathological confirmation is often required for definitive classification, particularly in cases with ambiguous cytological features [24].

Non-diagnostic samples constituted 33.80% of the total cases, with the highest proportions observed in lymph nodes, cutaneous and subcutaneous nodules, and miscellaneous. This finding highlights the impact of sampling limitations, including insufficient cellularity, excessive blood contamination, and poor slide preparation techniques. Given the considerable proportion of non-diagnostic cases, optimising sample collection and preparation protocols is crucial to enhancing cytological diagnostic utility [18].

No statistically significant association was found between the feline breed and the likelihood of neoplastic lesions, suggesting a relatively uniform distribution of neoplasia across breeds. This observation is consistent with existing epidemiological data indicating that while certain breeds may carry genetic predispositions, age and environmental exposures are more influential determinants in tumour development [51].

Similarly, no significant overall association was observed between sex and the occurrence of neoplastic lesions, suggesting that neoplasia affects male and female cats at comparable rates. However, a closer examination of specific tumour types revealed notable trends, with epithelial and melanocytic neoplasms more frequently diagnosed in females, while round cell and mesenchymal neoplasms were more prevalent in males. These findings align with prior research indicating that hormonal influences and genetic factors may contribute to differences in tumour distribution between sexes [51,52].

Certain tumour types are known to have sex-associated prevalence patterns in cats, with some neoplasms, such as mammary gland tumours, being significantly more common in females [30,47,52,53,54].

Some neoplasms, namely those of mesenchymal origin, have been reported to occur more frequently in male cats, although the underlying mechanisms remain incompletely understood [47,54]. Similarly, male cats were more frequently diagnosed with round cell neoplasms, like lymphoma, mast cell tumours, and plasma cell tumours. This aligns with prior research suggesting that male cats may have a higher risk of developing lymphoma, particularly in association with feline leukaemia virus (FeLV) infection, influenced by differences in immune function and retroviral susceptibility [47,52]. Beyond neoplasia, cytological diagnoses of inflammatory and non-neoplastic conditions did not demonstrate breed predisposition. However, previous research suggests that certain inflammatory conditions, such as eosinophilic granulomas, may be more common in breeds such as Siamese and other oriental breeds due to their unique immune responses [55,56]. The absence of a general sex predisposition to neoplasia in this study is consistent with previous large-scale epidemiological studies [47,52], which identify sex as a minor determinant of cancer risk, apart from hormonally driven tumours such as mammary carcinoma. However, the observed variations in tumour subtypes between sexes underscore the relevance of considering sex as a potential influencing factor during cytological evaluation.

The present study reinforces the well-documented association between advancing age and an increased prevalence of neoplastic conditions in felines. The cumulative effects of environmental carcinogens, chronic inflammation, and genetic mutations contribute to the heightened neoplastic risk observed in senior and geriatric patients [52,53]. Nonetheless, some studies challenge this association, suggesting that certain tumour types do not follow a clear age-related pattern and may even be more prevalent in younger individuals [51]. Although some tumours, such as lymphomas, may present in younger adult cats, particularly in those with retroviral infections like FeLV, widespread testing and vaccination led to a decline in this type of lymphoma, which are now more frequently diagnosed in older, FeLV-negative cats [57]. In contrast, inflammatory and non-neoplastic lesions were more common in younger cats, particularly those involving infectious processes or immune reactivity, like eosinophilic granulomas and pyogranulomatous inflammations.

Given the marked increase in neoplastic diagnoses in ageing cats, these findings underscore the importance of proactive diagnostic strategies, particularly in senior and geriatric patients. Regular cytological evaluation of suspicious lesions, coupled with imaging and histopathological confirmation where needed, can facilitate early detection and improve clinical outcomes [18,28,58,59]. In younger cats, clinicians should consider inflammatory and reactive processes as primary differential diagnoses before suspecting neoplasia. The highest odds ratio for neoplastic lesions was observed in geriatric cats (>15 years), who were nearly five times more likely to develop neoplasia compared to the reference group. This stark increase in cancer risk further supports the necessity for rigorous neoplastic surveillance in older felines [47,52,53,60].

## 5. Conclusions

The study underscores cytology as a minimally invasive, quick, and cost-effective technique for identifying neoplastic, inflammatory, and non-neoplastic lesions, especially in fluid-based, glandular, and mucous membrane samples with high diagnostic yields. However, the high rate of non-diagnostic samples (33.80%) calls for improved sampling protocols, operator training, and standardised cytological methods to achieve better diagnostic results.

Diagnostic performance varied with anatomical location, age, and sex of the subjects. Older cats showed a significantly higher rate of neoplastic conditions, with geriatric patients having a nearly fivefold increase in neoplastic diagnoses compared to younger counterparts, suggesting the need for age-specific screening. Although no link was found between breed and neoplasia, sex-based differences were noted; females had higher rates of epithelial and melanocytic neoplasms, whereas mesenchymal and round cell neoplasms were more common in males, indicating that genetic, hormonal, and environmental factors may influence neoplastic development in felines.

## Figures and Tables

**Table 1:** Occurrence of types of non-neoplastic/neoplastic lesions by age group.

		Non-Neoplastic		Neoplastic		Total
		*n*	% Within Non-Neoplastic/Neoplastic	*n*	% Within Non-Neoplastic/Neoplastic	*n*
**Age Groups**	≤1	218	89.71%	25	10.29%	243
	>1 to ≤2	99	78.57%	27	21.43%	126
	>2 to ≤4	134	75.71%	43	24.29%	177
	>4 to ≤7	202	71.89%	79	28.11%	281
	>7 to ≤10	179	57.01%	135	42.99%	314
	>10 to ≤15	242	48.79%	254	51.21%	496
	>15	42	42.00%	58	58.00%	100
	**Total**	1116	-	621	-	1737

**Table 2:** Occurrence of different types of neoplastic lesions categorised by sex and age.

			**Neoplastic Lesions**						
	**Round Cell**		**Mesenchymal**		**Epithelial**		**Melanocytic**		**Total**
	*n*	% within Sex	*n*	% within Sex	*n*	% within Sex	*n*	% within Sex	*n*
				**Sex**					
**Female**	122	31.12%	119	30.36%	149	38.01%	2	0.51%	392
**Male**	110	34.16%	138	42.86%	73	22.67%	1	0.31%	322
**Total**	232		257		222		3		714
				**Age**					
Age Groups (years)	*n*	% within Age Groups	*n*	% within Age Groups	*n*	% within Age Groups	*n*	% within Age Groups	*n*
≤1	21	84%	2	8%	2	8%	0	0%	25
>1 to ≤ 2	22	81.48%	1	3.7%	3	11.11%	1	3.7%	27
>2 to ≤4	19	44.19%	15	34.88%	9	20.93%	0	0%	43
>4 to ≤7	32	40.51%	26	32.91%	21	26.58%	0	0%	79
>7 to ≤10	36	26.67%	55	40.74%	43	31.85%	1	0.74%	135
>10 to ≤15	71	27.95%	85	33.46%	97	38.19%	1	0.39%	254
>15	15	25.86%	20	34.48%	23	39.66%	0	0%	58
**Total**	216		204		198		3		621

## Data Availability

The data presented in this study are available on request from the corresponding authors.
